# Tabula viva chirurgi: a living surgical document

**DOI:** 10.5830/CVJA-2015-081

**Published:** 2016

**Authors:** Marius J Swart, Gina Joubert, Jan-Albert van den Berg, Gert J van Zyl

**Affiliations:** Bloemfontein Mediclinic; Health Sciences Education, Faculty of Health Sciences, and Department of Practical Theology, Faculty of Theology, University of the Free State, Bloemfontein, South Africa; Department of Biostatistics, Faculty of Health Sciences, University of the Free State, Bloemfontein, South Africa; Department of Practical Theology, Faculty of Theology, University of the Free State, Bloemfontein, South Africa; Dean, Faculty of Health Sciences, University of the Free State, Bloemfontein, South Africa

**Keywords:** auto-ethnography, CABG, spirituality, surgery

## Abstract

**Aim:**

The purpose of this article is to present the results of a private cardiac surgical practice. This information could also serve as a hermeneutical text for new wisdom.

**Methods:**

A personal database of 1 750 consecutive patients who had had coronary artery bypass graft (CABG) surgery was statistically analysed. Mortality and major morbidity figures were compared with large registries. Risk factors for postoperative death were determined.

**Results:**

Over a period of 12 years, 1 344 (76.8%) males and 406 (23.2%) females were operated on. The observed mortality rate was 3.03% and the expected mortality rate (EuroSCORE) was 3.87%. After stepwise logistic regression, independent risk factors for death were urgency (intra-aortic balloon pump), renal impairment (chronic kidney disease, stage III), re-operation and an additional procedure. Apart from the 53 deaths, another 91 patients had major complications.

**Conclusion:**

Mortality and morbidity rates compared favourably with other international registries. Mortality was related to co-morbidities. This outcome contributes to a hermeneutical understanding focusing on new spiritual wisdom and meaning for the surgeon.

## Abstract

Cardiac surgical risk models for postoperative mortality in the South African context do not exist. An effort to set up a registry or database for cardiac surgery was unsuccessful.^1^ Local surgeons have to rely on results from outside South Africa to compare their results. Risk models established in Europe or the United States of America cannot necessarily be applied.^2^ Patient profiles differ from region to region.

As an alternative, one can use the published results from South African units as a yardstick. However, the reporting of outcome after coronary artery bypass graft surgery (CABG) is not common in South Africa. Between 1961 and 2009, five articles informed on general outcome after CABG surgery.^3^ In 1972 Wentworth Hospital, Durban, described 20 patients who had CABG surgery.^4^ In 1979 the Department of Cardiothoracic Surgery at the University of the Free State published their results on the first 50 CABG cases,^5^ and followed it up with the first 100 patients in 1983.^6^ Groote Schuur Hospital contributed with 204 patients that were operated on between 1976 and 1978.^7^ In 1982, Tygerberg Hospital added their 118 cases operated on between 1978 and 1980.^8^ None of these publications referred to mortality risk.

After the literature search was repeated in 2014, a further two reports on CABG outcome were found. Both were small in number and both emphasised a specific subset of patients and not CABG in general.^9^,^10^

One other author also contributed with published results after CABG. A randomised, double-blind study from a single centre, on the effect of aprotonin on cardiac surgery (50 CABG cases and 50 valve cases), was conducted in the early 1990s at Groote Schuur Hospital.^11^ This study had exclusion criteria and was not representative of a cardiac surgical practice.

The first local comparison of outcome with a risk score (EuroSCORE) appeared in the European Journal of Cardiothoracic Surgery as a letter to the editor in 2004.^12^ Only 574 cases were involved, compared to the much larger numbers of most European or American studies. The impact of impaired renal function as calculated by the simplified modification of diet in renal disease (sMDRD) and the metabolic syndrome on CABG outcome was reported in two separate articles.^13^,^14^ These studies excluded additional procedures in conjunction with CABG and did not signify a true registry type of database.

Evaluating surgical outcome is common among cardiac surgeons.^15^ Looking at mortality and morbidity rates is one way of assessing outcome; however, a new interpretation of such data could also lead to a different form of knowledge or wisdom. As part of a spiritual reflection on negative outcomes after CABG in an auto-ethnographic study, the database of one of the authors (MJS) was presented as a personal document or text for re-interpretation.^16^

The science of text interpretation is also known as hermeneutics. Each human being can be read as a living document, similar to a historical text or piece of art. According to Gerkin, this reading of a living document could be an effort to reconcile experience with theological language,^17^ hence the title of this article: Tabula viva chirurgi: a living surgical document. Theological language has a spiritual undertone. In the words of Henri Nouwen, this spirituality should be a movement from the restlessness of loneliness to the restfulness of solitude.^18^

The aim of this article was to acknowledge the negative outcomes of some patients after CABG, to articulate it and to interpret it. This should present insight into a private cardiac surgical practice in South Africa where there is generally a lack of published data, but also accentuate the possibility of a spiritual gain from such an endeavour by obtaining new wisdom.

## Methods

All patients who had CABG surgery done consecutively by one surgeon (MJS) between November 2000 and November 2012 in the Mediclinic Hospital, Bloemfontein, were included. The information was obtained from a personal and ongoing database. Almost all the patients were operated with conventional cardiopulmonary bypass and cardiac arrest. The Ethics Committee at the Faculty of Health Sciences, University of the Free State, approved the study as part of a thesis.

Mortality was defined as death while in hospital. This is in line with the EuroSCORE II as well as the Cleveland Clinic.^19^,^20^ Pre-operative renal function was determined with the sMDRD formula. Impairment was defined as a calculated glomerular filtration rate (GFR) of less than 60 ml/l/1.73 m^2^ or chronic kidney disease stage III (CKD III).

Major postoperative morbidity as reported by the Society of Thoracic Surgeons (STS) implies re-exploration, prolonged ventilation (> 48 hours), permanent stroke, renal failure and deep sternal infection.^21^ For this study, renal failure after surgery was not defined on the basis of a doubling in serum creatinine value, but as new dialysis. The STS has subsequently adjusted its definitions for prolonged ventilation and renal failure.^22^ All patients who had rewiring after dehiscence of the sternum while in hospital, as well as within six weeks after discharge, were considered to have deep mediastinal infection. In addition, gastrointestinal complications, postoperative bleeding, the use of homologous blood products and length of hospital stay (LOS) were also investigated.

Postoperative care was done by the surgeon in conjunction with the nursing staff. In general the physicians get involved once the patient develops multi-organ failure.

The additive EuroSCORE of 1999 was used to calculate the risk for operative mortality for each patient.^23^ Towards the end of the study time period, the EuroSCORE II became available and was introduced into the practice, but this was not applied to this study.^19^

## Statistical analysis

The statistical analysis was done by the Department of Biostatistics of the Faculty of Health Sciences at the University of the Free State. Results were summarised by calculating means with standard deviations or percentiles (numerical variables), and frequencies and percentages (categorical variables). Individual possible risk factors’ relationship with mortality was investigated using chi-squared or Fisher’s exact tests. Significant univariate risk factors were included in a logistic regression.

## Results

A total of 1 750 patients had a CABG done. Of these patients, 122 (7.0%) had an additional procedure Table 1.. Males were in the majority at 76.8%, with females at 23.2%. The age range was between 20 and 87 years old. The median age for males was 61 years and for females 64 years. Table 2. depicts a profile of this population of CABG patients.

**Table 1:** Additional procedures to the CABG

*Additional procedure*	*Number*	*Percentage (n = 1 750)*
Aorta dissection (intra-operative complication)	1	0.1
Aortic valve replacement	51	2.9
Aortic valve + mitral valve replacement	3	0.2
Aortic valve + mitral valve replacement + Maze	1	0.1
ASD	1	0.1
ASD + Maze	1	0.1
Biopsies for carcinoma	3	0.2
Left ventricular aneurysm	2	0.1
Left ventricular aneurysm + Maze	1	0.1
Left ventricular rupture	1	0.1
Maze	6	0.3
Mitral valve repair	15	0.9
Mitral valve repair + Maze	2	0.1
Mitral valve replacement	29	1.7
Mitral valve replacement + Maze	4	0.2
VSD (ischaemic)	1	0.1

ASD, atrio-septal defect; VSD, ventriculo-septal defect.

**Table 2:** Profile of CABG patients

*Profile*	*Number*	*Percentage of total*
Females	406	23.2
Males	1344	78.8
≤ 39 years	39	2.2
40–49 years	204	11.7
50–59 years	505	28.9
60–69 years	604	34.5
70–79 years	363	20.7
≥ 80 years	35	2.0
Diabetes mellitus	442	25.3
Urgent (IABP/ventilator)	312	17.8
Renal impairment (CKD III)	376	21.7
Re-operation (2nd, 3rd, 4th operation)	196	11.2
Additional procedure	122	7.0

CKD, chronic kidney disease; IABP, intra-aortic balloon pump.

During hospitalisation, 53 patients (3.03%) died. A one-word cause of death was given for each patient who died Table 3.. The expected mortality rate was 3.87% (69 patients). The observed/expected mortality rate (O/E) was 0.78. In the original EuroSCORE population the mortality rate was 4.80%.^23^

**Table 3:** Reason for death

*Reason for death*	*Number of deaths (%)*
Cardiac (death in theatre)	5 (9.4)
Surgical bleed	1 (1.9)
Sudden and unexplainable	5 (9.4)
Stroke	2 (3.8)
Brain dead	1 (1.9)
Inflammatory conditions (DIC, IE, sepsis, SIRS)	7 (13.2)
Gastrointestinal	3 (5.7)
Organ failure (cardiac, respiratory, renal, MOF)	28 (52.8)
Pining away	1 (1.9)

DIC, diffuse intravascular coagulopathy; IE, infective endocarditis; MOF, multiorgan failure; SIRS, systemic inflammatory response syndrome.

Risk-adjusted mortality (RAM) allows individual surgeons to compare their results within a larger group of patients.^24^ The RAM for this series was therefore 3.74% (0.78 × 4.80%), which is less than the EuroSCORE cohort. Isolated CABG (no additional procedure done with the CABG) had an observed mortality of 2.21% and an expected mortality of 3.63% with an O/E of 0.61. More than a quarter (26.3%) of the patients was considered high risk for operative mortality, i.e. EuroSCORE ≥ 6.0.

## Univariate analysis

The Working Group Panel on the Cooperative CABG Database Project was used as a reference point.^25^ Seven core risk factors for mortality were identified during the mid-1990s. These risk factors were age (younger than 70 years, and 70 years or older were arbitrarily selected by the surgeon), gender, re-operation, left main stem, low ejection fraction (40% was applied as the cut-off point), urgency, and number of coronary artery systems involved. Data on the last factor are not available for this series.

From Table 4. it is clear that of these factors, the female gender was not significantly associated with operative mortality rate. As expected, patients with chronic obstructive pulmonary disease (COPD) were at higher risk (223 patients with 5.8% mortality rate, compared to 2.6% for the other patients) (p = 0.0090). About one-fifth (21.7%) of the patients had renal impairment (CKD III) based on the calculated GFR. Their mortality rate was 6.7% compared to 2.1% for the rest (p < 0.0001). Other potential risk factors that were not significantly associated with operative mortality included hypertension, diabetes mellitus and body mass index (BMI ≥ 30 kg/m^2^), not even in combination, as in the metabolic syndrome.

**Table 4:** Core risk factors^25^

*Risk factor*	*Mortality/total*	*Mortality (%)*	*p-value*
< 70 years	31/1 352	2.3	
≥ 70 years	22/398	5.5	0.0009
Male	46/1 344	3.4	
Female	7/406	1.7	0.0801
1st sternotomy	35/1 554	2.3	
2nd sternotomy	14/179	7.8	
3rd sternotomy	4/15	26.7	
4th sternotomy	0/2	0.0	< 0.0001
No left main stem	34/1 420	2.4	
Left main stem	19/330	5.8	0.0013
LVEF ≥ 40%	41/1 652	2.5	
LVEF < 40%	12/98	12.2	< 0.0001
Home/ward	14/570	2.5	
CCU	21/868	2.4	
IABP	12/299	4.0	
Ventilator/lab	6/13	46.2	< 0.0001

CCU, coronary care unit; IABP, intra-aortic balloon pump; lab, catheter laboratory; LVEF, left ventricular ejection fraction.

A perception that the patients of some referring cardiologists are at higher risk was tested Table 5. Six cardiologists are or were involved at the Mediclinic, Bloemfontein, at some point. One of the six ‘cardiologists’ actually represents a number of cardiologists, each with only a small number of cases. Cardiologist B had a higher risk score and therefore also a higher mortality rate than A, D and E. Cardiologists B and C (the latter had only 58 cases) did not differ significantly regarding mortality rate.

**Table 5.** Cardiologist’s mortality/risk and contribution to the cohort

*Cardiologist*	*Mortality/ total*	*Mortality (%)*	*EuroSCORE*	*Contribution to the total (%)*
A	12/484	2.5	3.57	27.6
B	19/335	5.7	4.76	19.1
C	0/58	0.0	4.36	3.1
D	5/309	1.6	3.69	17.7
E	11/431	2.6	3.55	24.6
F	6/133	4.5	4.17	7.6

## Stepwise logistic regression

All the significant risk factors were used to establish a model of factors that are significantly associated with mortality in a multivariate logistic regression. Four risk factors could be considered independent risk factors: urgency (intra-aortic balloon pump; IABP), renal impairment (CKD III), re-exploration, and an additional procedure Table 6..

**Table 6:** Significant risk factors in logistic regression with odds ratio

*Risk factor*	*OR*	*95% CI*
Urgency (IABP)	2.21	1.13–4.32
Renal impairment (CKD III)	2.58	1.44–4.65
Re-operation	4.31	2.32–8.00
Additional procedure	7.14	3.60–14.18

CI, confidence interval; CKD, chronic kidney disease; OR, odds ratio; IABP, intra-aortic balloon pump.

## Morbidity

Besides the 53 deaths, 115 patients had a major complication during their initial stay in hospital; however, 31 of these patients also had a major complication after the operation and then died (22 patients died without an official major complication). A further seven patients were included as morbidity after they had been discharged, but were re-admitted with sternal dehiscence. The combined mortality and major morbidities involved 144 patients (8.2%). Table 7. illustrates the prevalence of major morbidities. Some patients (23 + 5) had more than one major complication and their mortality rate was 50%. The low mortality rate (1.0%) associated with no major complication is obvious Table 8..

**Table 7:** Major morbidities

*Complication*	*Number of patients (%)*
Re-exploration	32 (1.8)*
Prolonged ventilation	36 (2.1)
Renal failure	32 (1.8)
Permanent stroke	20 (1.1)
Sternal dehiscence	17 (1.0)

The percentage was calculated from 1 745 patients as five died in theatre.

*31 for bleeding and one instrument that was left behind.

28 patients had more than one major complication (Table 8).

**Table 8:** Patients with associated major complications

*Number of patients*	*With major complication*	*Mortality (%)*
94	Single	17 (18.1)
23	Double	12 (52.2)
5	Triple	2 (40.0)
1 623*	None	17 (1.0)

*Five patients died in theatre and had no major complications.

The number of patients was 1 750 (5 + 94 + 23 + 5 + 1 623).

The number of deaths was 53 (5 + 17 + 12 + 2 + 17).

Gastrointestinal complications are not often regarded as major complications, yet some are very serious. Twenty-one such patients (1.2%) were identified, of whom 11 had a gastroscopically diagnosed peptic ulcer; four presented with active bleeding and six had laparotomies. This finding was despite the routine use of a proton pump inhibitor. The laparotomies were done for bowel ischaemia (three), bowel obstruction (two) and ulcer perforation (one). There were four (19.0%) deaths among these 21 patients, two with bleeding and two with ischaemia.

Re-exploration for mediastinal bleeding is considered a major complication and was necessary in 31 patients (1.8%). Mediastinal drainage was measured over 48 hours. The average bleeding plus one standard deviation was considered major bleeding. A calculated volume of 1 070 ml per 48 hours was therefore considered important. There were 180 (10.3%) such patients. Table 9. illustrates the association between bleeding and mortality. The significant difference between 9.4 and 2.0% had a p-value of < 0.0001.

**Table 9:** Outcome associated with mediastinal drainage

*Drainage*	*Number (%)*	Mortality (%)*	*Re-exploration (%)*
≥ 1 070 ml	180 (10.3)	17 (9.4)	29 (16.1)
< 1 070 ml	1 565 (89.7)	31 (2.0)	3 (0.2)**

*Five patients died in theatre (total mortality 53).

**The re-explorations were in two patients who drained 950 and 975 ml and the third patient had to be re-opened to remove a surgical instrument.

Cardiac surgery is an important consumer of homologous blood products. Of these 1 750 patients, only 404 (23.2%) patients actually received blood products. Almost a quarter of this surgical population depended on the blood bank for red blood cells (RBC), plasma and/or platelets. The close association between risk, mortality and blood bank usage is demonstrated in Table 10.. The risk and outcome between the group with three and more units of RBC and those with less differed considerably (p < 0.0001).

**Table 10:** Risk, mortality and blood bank usage

Blood bank usage*	*EuroSCORE*	*Mortality (%)*
No bank blood (n = 1 346)	3.43	23 (1.7)
Bank blood (n = 404)	5.33	30 (7.4)
Only 1–2 units RBC (n = 222)	4.95	6 (2.7)
≥ 3 units RBC (n = 123)	6.05	20 (16.3)

RBC, red blood cells. *Bank blood includes red blood cells, plasma and/or platelets.

## Length of stay

The average length of stay (LOS) of the 1 697 patients who left hospital was 6.0 days (2–83 days, median 5). LOS is an indication of recovery and that should correlate with age and risk for mortality. Table 11. and Table 12. confirmed this. For isolated CABG (n = 1 628 patients) 63.8% of patients stayed five days and less in hospital, whereas only 2.2% stayed longer than two weeks.

**Table 11:** Length of stay and age

*Total group (n = 1 697)*	*Median (days)*
Age (years)	5
≤ 39 (n = 39)	4
40–49 (n = 203)	4
50–59 (n = 495)	5
60–69 (n = 584)	5
70–79 (n = 346)	6
≥ 80 (n = 30)	9

**Table 12:** Length of stay and EuroSCORE

*Total group (n = 1 697)*	*Median (days)*
EuroSCORE	5
≤ 2 (n = 630)	5
3–5 (n = 644)	5
6–9 (n = 365)	6
≥ 10 (n = 58)	10

## Discussion

The exposition of such surgical outcome data might appear like basic auditing of a practice, yet one should always be attuned to more wisdom. The scientist looks for wisdom of theoria and the surgeon evaluates for wisdom of technē. A practical wisdom obtained with a process of hermeneutics against a certain traditional background, such as faith, is referred to as phronēsis.^26^

Mortality rate is one way of assessing outcome, but favourable mortality rates could also indicate limited morbidity and even long-term survival.^27^ Registries provide a more accurate picture of mortality as an outcome. In fact, published articles underrepresent mortality rate up to 50% lower than a database.^27^ A statistical comparison between the local outcome and records from both sides of the Atlantic Ocean and Japan was not possible and the reader is left with a visual comparison. Table 13. displays such a comparison with other databases.

**Table 13:** Comparison of isolated CABG mortality with other databases

*Databases*	*Year*	*Number*	*Mortality (%)*
Tabula viva chirurgi	2000–2012	1 628	2.21
EuroSCORE^23^	1998	12 103	3.40
STS^22^	2000–2006	774 881	2.30
JACVSD^35^	2000–2005	7 133	2.72

JACVSD, Japan Adult Cardiovascular Surgery Database; STS, Society of Thoracic Surgeons.

Mortality could also be defined as death within 30 days, even if the patient had been discharged. Locally, the majority of patients are from outside the city where surgery is performed and follow up is limited. To balance the odd patient who might have died at home within 30 days are those cases where the patient died after several weeks in hospital with, for example, respiratory failure or after a second operation. They were all considered as primary cardiac surgical mortalities. Decanting refers to transferring a critical patient to a second facility and so the mortality or morbidity is erased.^28^ This was not and is still not the practice in Bloemfontein.

The determination of the aetiology of death is not simple and could differ from surgeon to surgeon. In the Tabula viva chirurgi it seems most patients died due to a non-cardiac system failure Table 3. A post mortem is done only in cases of death in theatre or in cases where the patient has not woken up. That being said, a routine post mortem is not always clarifying.^29^ The phase-of-care mortality analysis (POCMA) identifies an identifiable trigger for a fatal course.^30^ These five phases are pre-operative, the operation itself, while the patient is in intensive care, in the ward, and during the discharge phase. Such a seminal event leading to death was found in 35% during the pre-operative phase by Shannon et al.^30^

For the local study, a POCMA was not done. In practice, it means an error in selection, an error in procedure or an error in care. Hence co-morbidities and mortality risk factors are part of the selection criteria. Determining those factors was important for the surgeon, as every death or major complication emphasises the surgeon’s feeling of responsibility or loneliness. This is an emotional response, but it could progress to spiritual reflection.

Some of the risk factors of the 1990s are even now important contributors to the risk for death. In this particular study, female gender was not a risk for mortality, although female patients had a higher EuroSCORE than the males (4.7 vs 3.6). In fact, male gender was almost a mortality hazard (p = 0.0801). In the EuroSCORE II, female gender still contributes to death after surgery, although this contribution is small.^19^ Others claim that it is not the gender per se, but rather the associated co-morbidities associated with the female gender.^31^

The finding that the referring cardiologist contributes to the risk and therefore mortality rate was expected Table 5.; however, it was not significant in the stepwise logistic regression. Cardiologist B referred more patients with an additional procedure (40.2% of the total of 122). Cardiologist C had only one such patient, but then C presented 11.0% of patients with an IABP, whereas C’s contribution to the total number of cases was only 3.1%. In the EuroSCORE, IABP is a significant factor for risk/mortality. It could be argued that Cardiologist B had a lower threshold to refer patients with a higher risk for surgery in an attempt to treat the patient. It could also mean that Cardiologist B has a more aggressive interventional approach towards lowerrisk patients. It is hoped that this also attests to confidence in the surgical team.

The significant risk factors from the Tabula viva chirurgi after logistic regression differed from the original Working Group Panel on the Cooperative CABG Database Project.^25^ Only re-operation and urgency remained as risk factors. Three of the five theatre deaths were re-operations and the patient who bled to death also had a second operation. The other deaths among the re-operation group of patients had the same mixture of reasons for death as the rest Table 3.. In a large series of 1 521 re-operations, the mortality rate was 9.7% (in the Tabula viva chirurgi re-operations had a mortality rate of 9.2%).^32^ Pre-operative renal impairment (CKD III in particular) and an associated procedure were not investigated in the Working Group during the 1990s.

If all the patients with one or more of these four significant risk factors were excluded, the mortality rate would drop to seven of 963 patients (0.73%), with an expected mortality rate of 2.40% (EuroSCORE). Another way of confirming the contribution of risk factors is to look at the observed mortality rate of those patients with an expected mortality rate of 0% (i.e. EuroSCORE 0). One individual of 237 patients died (0.42%). In the recent EuroSCORE II, the lowest possible risk of mortality is in any case 0.5%.

The impact of major morbidity on mortality is illustrated in Table 8.. Not all patients who bleed more than expected require re-exploration, nevertheless excessive drainage is stressful to the surgeon. To take patients back to theatre for bleeding/ tamponade puts severe strain on the surgeon. It is not only a utilisation of resources (human and financial), but the bleeding, re-opening of the chest and subsequent blood transfusion put the patient at further risk. It is comforting to realise that a unit such as Cleveland takes 3.0% of patients back to theatre.^33^

A Swedish study reported on 2 000 CABG patients,^34^ with a re-exploration rate of 4.9% and, interestingly enough, 10.0% (similar to the Tabula viva chirurgi) drained more than 1 000 ml, measured over 12 hours. In the Tabula viva chirurgi, the measurement was done over 48 hours. Although 36 patients were ventilated for longer than 48 hours, 15 patients were ventilated for respiratory reasons and 21 because of a suppressed level of consciousness.

Renal failure in particular needs to be addressed. As an isolated complication, renal failure was found in 14 patients, and another 15 had renal failure combined with mechanical ventilation. Three patients had a third complication. Of these 32 (1.8%) patients, 25 required dialysis (seven patients died before renal intervention). Prolonged mechanical ventilation with renal failure (dialysis) is a lethal combination as 11 of 15 such patients died. Only one of 10 patients who required postoperative dialysis as an isolated major complication, died. Two-thirds of the patients with renal failure were pre-operatively CKD III.

Both Cleveland Clinic^34^ and the Japan Adult Cardiovascular Surgery Database (JACVSD)^35^ consider renal failure as new dialysis. The prevalence of renal failure (new dialysis) in the JACVSD was 3.18%. The STS defines renal failure as a critical rise in serum creatinine.^21^,^22^ In the Tabula viva chirurgi, 144 (8.2%) patients increased their basal creatinine level by 50% or higher postoperatively. This remains a sign of renal damage.

It is important to realise that three-quarters of patients (Tabula viva chirurgi) are operated on in hospital directly after their coronary angiogram and were therefore exposed to contrast medium. The referring cardiologist is usually concerned about unstable angina and critical coronary artery anatomy. These patients are all at risk for renal injury and even more so after the surgery that might follow.^36^ Surgery within five days of angiography has an odds ratio of 1.82 to lead to renal impairment.^37^ This is regardless of a pre-operative glomerular filtration rate < 60 ml/l/1.73 m^2^, and cardiopulmonary bypass duration, which also affects renal performance.^37^

Stroke is a shattering complication. Apart from it being a blow to the patient and to those caring for him/her, a stroke in particular contributes to this burden of liability surgeons might experience, not only from a medico-legal perspective, but also from a spiritual viewpoint. Stroke occurred in 20 patients (1.1%), 11 as an isolated complication, but in another nine as an associated major adverse event Table 6.. Four patients died, therefore a mortality rate of 20%. This stroke rate is also in accordance with the STS and JACVSD, 1.4% and 1.5%, respectively, but both registries were for isolated CABG.^22^,^35^ The unit at Emory University reports a prevalence of 2.2% with an almost similar mortality rate of 22.5%.^38^

Deep sternal sepsis is another major complication of the STS. The Centres for Disease Control and Prevention provide criteria for the diagnosis of deep sternal sepsis.^39^ In the local series, dehiscence of the sternum was documented for deep sternal sepsis. Ten patients were taken back to theatre for rewiring during the initial hospital stay and another seven were discharged, but were re-admitted within six weeks. Two of the patients were also mechanically ventilated and one of them had a stroke as a third major complication. None of these 17 (1.0%) patients died. Their median BMI was 29.4 kg/m^2^ and those with intact sternums had a BMI of 28.4 kg/m^2^.

The presence of COPD was found in 29% of patients with sternal dehiscence compared with 12% among those with a sternum in one piece. In a large Finnish study, 70% of patients with deep sternal sepsis were taken to theatre. This represents a prevalence of 0.8%.^39^ Patients are under the impression that the chest bone does not heal. A South African study investigated the general picture of post-CABG patients after six weeks and found six patients of 179 with no healing of the chest bone. Two of them had already had a second attempt in hospital to approximate the sternum.^40^ At least three others had risk factors such as prolonged mechanical ventilation and heart failure with pneumonia.

Most patients in our series were not from Bloemfontein, where the operation was done. They had to be well enough to travel back to whence they came, where health facilities are often limited. In general, patients are discharged only when the risk of re-admission is low. According to the STS registry, 51.2% of patients had an LOS of less than six days and 5.6% were hospitalised for more than two weeks.^22^ As far as isolated CABG is concerned, the hospital LOS compared well with the STS.

## Limitations

Larger numbers have more statistical power, but smaller numbers are the reality of a typical South African cardiac unit. In the private sector one finds units with a single surgeon and many with only a few surgeons per unit. Although the surgeon used transparent definitions, there was still a personal interpretation. The additive EuroSCORE was used but this probably underestimated the real mortality risk. The logistic EuroSCORE was perhaps more accurate for the higher-risk patient, especially in a low-volume practice.^41^ It has been stated before that 26.3% of patients fell into a higher-risk category. Low-risk patients did very well, with an O/E of < 0.61 and even less.

To determine a unit’s care of patients with major complications is a good way of assessing performance. The fewer the number of patients that die with a major complication, the better the unit performs. Those are the patients who failed to be rescued (FTR). A Canadian unit reported on 5 000 various open cardiac surgical cases over five years,^42^ where the mortality rate was 3.6%. Ten important complications were associated with 92% of the deaths. Their calculated FTR was 19.8%. In other words, in spite of major complications, 80.2% of patients survived. A FTR for the Tabula viva chirurgi would have been a good indication of how Mediclinic, Bloemfontein, fares as far as the care of patients with major complications is concerned, but we lacked data for this to be calculated. Table 8 might give some indication of the outcome of the patients with one or more of the five major complications. Of the patients with one or more major complications, 25% died.

## Conclusion

It was not the intention to present these data as a benchmark for the South African context, but it opens a window on a private cardio-surgical practice in South Africa. The outcomes are in line with those of established units and databases all over the world. This is wisdom related to the scientific and technical aspects associated with cardiac surgery and care. These findings also bring a new wisdom to the fore, which allows the surgeon to move from loneliness to solitude as a spiritual movement. The disappointments of negative surgical outcomes should move on to make the surgeon’s responsibilities a vocation instead of a burden. This allows the surgeon to provide hospitality as an alternative to hostility towards the patient.^18^ Such a spiritual experience should also have spiritual transformation as an outcome.^43^ As part of creation, physiological limits exist and surgical outcome is based on these limits. Mortality and morbidity are time and again linked to co-morbidity and surgical risk. This has implics for the pastoral and spiritual care of the sick.
